# Traditional Chinese Medicine: A promising strategy to regulate inflammation, intestinal disorders and impaired immune function due to sepsis

**DOI:** 10.3389/fphar.2022.952938

**Published:** 2022-09-16

**Authors:** Xu-Hua Wang, Ding-Qiao Xu, Yan-Yan Chen, Shi-Jun Yue, Rui-Jia Fu, Lu Huang, Yu-Ping Tang

**Affiliations:** Key Laboratory of Shaanxi Administration of Traditional Chinese Medicine for TCM Compatibility, State Key Laboratory of Research & Development of Characteristic Qin Medicine Resources (Cultivation), Shaanxi Key Laboratory of Chinese Medicine Fundamentals and New Drugs Research, Shaanxi University of Chinese Medicine, Xi’an, China

**Keywords:** sepsis, intestinal microbiota, traditional Chinese medicines, gut barrier, immune system

## Abstract

Sepsis is described as a dysregulation of the immune response to infection, which leads to life-threatening organ dysfunction. The interaction between intestinal microbiota and sepsis can’t be ignored. Furthermore, the intestinal microbiota may regulate the progress of sepsis and attenuate organ damage. Thus, maintaining or restoring microbiota may be a new way to treat sepsis. Traditional Chinese medicine (TCM) assumes a significant part in the treatment of sepsis through multi-component, multi-pathway, and multi-targeting abilities. Moreover, TCM can prevent the progress of sepsis and improve the prognosis of patients with sepsis by improving the imbalance of intestinal microbiota, improving immunity and reducing the damage to the intestinal barrier. This paper expounds the interaction between intestinal microbiota and sepsis, then reviews the current research on the treatment of sepsis with TCM, to provide a theoretical basis for its clinical application.

## GRAPHICAL ABSTRACT

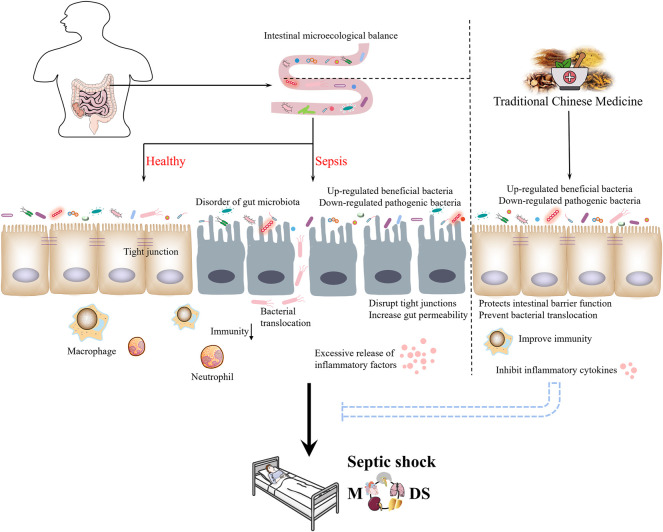

## 1 Introduction

Worldwide, sepsis continues to pose a serious public health concern. According to a third international consensus, sepsis is a systemic inflammatory response caused by infection, which leads to life-threatening organ dysfunction (Singer et al., 2016). Sepsis, which may result in septic shock and multiple organ dysfunction syndrome (MODS), kills over 5.3 million people worldwide per year (Dellinger et al., 2013; Fleischmann et al., 2016). Consequently, sepsis has been designated as one of the global health priorities by the World Health Organization (Fay et al., 2019). Recent studies reported that COVID-19 infection may cause life-threatening sepsis (Liu, et al., 2020b; Wiersinga et al., 2020). Among the 218,184 patients hospitalized for COVID-19, the overall pooled sepsis prevalence was estimated at 51.6% (Karakike et al., 2021). Sepsis, as a major global health issue, needs intensive research, which is also important for improving the clinical care of patients with COVID-19.

The intestinal microbiota is known as the body’s “second genome” and “forgotten organ”, which plays a crucial role relevant to human health and disease (Zhou et al., 2021). The human gut is a complex ecosystem, with the intestinal microbiota of a healthy individual consisting of approximately 400–500 species of bacteria (Zakerska-Banaszak et al., 2021), and they vary across individuals. These intestinal microbiotas mainly have a place with four significant phyla, namely *Firmicutes*, *Bacteroides*, *Actinobacteria*, and *Proteobacteria*, account for 90% of the intestinal microbiota (Rinninella et al., 2019; Álvarez-Mercado et al., 2019). When an infection occurs, our natural microbiota is our first line of protection. The pathogenesis of sepsis is complex, which is closely linked to a dysregulated inflammatory response and immune dysfunction, and the intestinal microbiota participants in this important process. The intestinal system is protected from colonization by exogenous microbiota and possibly harmful resident microbiota by the intestinal epithelial barrier, immune system, and intestinal microbiota (Kamada et al., 2013). Furthermore, previous examinations have indicated that the intestinal microbiota can influence damage reactions in organs outside of the gastrointestinal tract (Gong et al., 2019). This shows that intestinal microbiota has an important role in the occurrence and development of sepsis, which may represent a new frontier in sepsis treatment.

Sepsis has always been a focus of interest for researchers because of its extremely high incidence and mortality rates and the lack of ideal treatment in clinical medicine. The current western medicine treatment for sepsis is mainly antibiotics and supportive care (Varkouhi et al., 2020). One of the most frequently used medications in the treatment of sepsis in clinical applications is antibiotics. It can alter the intestinal microbiota significantly and negatively impact sepsis outcomes (Dethlefsen et al., 2008; Adelman et al., 2020). For example, one study showed that the use of antibiotics will affect the intestinal microbiota, leading to an imbalance in the intestinal microbiota that can persist for up to 6 months (Faura et al., 2021). According to the clinical manifestations of sepsis, it belongs to the traditional Chinese medicine (TCM) “febrile diseases” category (Lin S. J. et al., 2013). TCM treatment for sepsis mainly includes the methods of clearing heat and detoxifying toxins (Qingrejiedu), promoting blood circulation and removing blood stasis (Huoxuehuayu), and promoting the recovery of physiological function (Fuzhengguben). TCM, as a strong supplement to antibiotic drugs, has been included in the normal treatment of sepsis (Liu L. et al., 2020), such as XueBiJing injection (XBJ), ShenFu injection, ShengMai formula, Xuanbai Chengqi decoction (XBCQ), and Qingwen Baidu decoction (QWBD), etc (Table 1). TCM has been proved to be effective in the treatment of sepsis and has advantages in improving intestinal microbiota disorders and maintaining intestinal homeostasis. In this review, we discussed the intestinal microbiota and sepsis relationship and the TCM in modulating intestinal microbiota for the therapy of sepsis.

**TABLE 1:** Formulation composition and pharmacological actions of representative TCM.

Prescription	Formulation composition	Pharmacological actions	References
XueBiJing Injection	*Carthamus tinctorius* L. (Honghua), *Paeonia lactiflora* Pall. (Chishao), *Salvia miltiorrhiza* Bunge (Danshen), Conioselinum anthriscoides ‘Chuanxiong’ (chuanxiong), *Angelica sinensis* (Oliv.) Diels (Danggui)	down-regulated the inflammatory response (IL-6, TNF-α, and IL-10, *etc*.) and the activation of signaling pathways (NF-κB, MAPK, and PI3K/Akt) initiated by Pam3CSK4	Li T. et al., 2020; Li et al. 2016
Shenfu Injection	*Panax ginseng* C. A. Mey. (Hongshen), processed *Aconitum carmichaeli* Debeaux (Fuzi)	inhibits MEK and ERK signal pathways, reduces apoptosis, and alleviates sepsis-induced inflammation and mitochondrial damage	Chen et al., 2020b; Xu et al., 2020
Qingwen Baidu Decoction	*Rehmannia glutinosa* (Gaertn.) DC. (Dihuang), Cornu Bubali (Xijiao), *Coptis chinensis* Franch. (Huanglian), *Gardenia jasminoides* J.Ellis (Zhizi), *Platycodon grandiflorus* (Jacq.) A. DC. (Jiegeng), *Scutellaria baicalensis* Georgi (Huangqin), *Anemarrhena asphodeloides* Bunge (Zhimu), *Paeonia lactiflora* Pall. (Chishao), *Scrophularia ningpoensis* Hemsl. (Xuanshen), *Forsythia suspensa* (Thunb.) Vahl (Lianqiao), *Lophatherum gracile* Brongn. (Danzhuye), *Glycyrrhiza glabra* L. (Gancao), *Paeonia × suffruticosa* Andrews (Danpi), Gypsum Fibrosum (Shigao)	reducing the level of inflammation, inhibited the activation of NF-κB and STAT3 signal pathways	Yu et al., 2014; Zheng et al., 2020
Huanglian Jiedu Decoction	*Phellodendron chinense* C. K. Schneid. (Huangbai), *Scutellaria baicalensis* Georgi (Huangqin), *Gardenia jasminoides* J. Ellis (Zhizi), *Coptis chinensis* Franch. (Huanglian)	the cholinergic anti-inflammatory pathway was activated and the HMGB-1/TLR4/NF-κB signal pathway was inhibited, resulting in reduction of production of IL-6 and TNF-α	Xu et al., 2017; Chen et al., 2017
Xuanbai Chengqi Decoction	*Rheum palmatum* L. (Dahuang), *Gypsum Fibrosum* (Shigao), *Prunus armeniaca* L. (Xingren), *Trichosanthes kirilowii* Maxim. (Gualou)	modulating the gut microbiota, restoring the gut barrier, and downregulating inflammatory responses	Mu et al., 2021

TNF-α, tumor necrosis factor alpha; IL-6, Interleukin-6; IL-10, Interleukin-10; LPS, lipopolysaccharide; Pam3CSK4, a synthetic tripalmitoylated lipopeptide mimicking bacterial lipoproteins.

## 2 The relationship between sepsis and intestinal microbiota

### 2.1 Intestinal microbiota disruption predisposes to sepsis

Two epidemiological studies have provided indirect evidence that disruption of the intestinal microbiota predisposes to sepsis. The first study, which included more than 10,000 Medicare recipients, found that patients with *Clostridium difficile* infection (CDI) were more likely to be admitted with severe sepsis than without CDI patients (Prescott et al., 2015). The second study, which looked at more than 12 million patients, found that those who were exposed to antibiotics on admission were more likely to be readmitted within 90 days after recovery, compared with those who were not exposed to antibiotics (Baggs et al., 2018). Furthermore, the decreased diversity of the intestinal microbiota also has an impact on sepsis in animal models. Cecal ligation and puncture (CLP) was performed on two groups of mice with the same genetic background to create sepsis models. When the microbiota of the two groups were significantly different, mice with greater alpha diversity had a better chance of survival than mice with less alpha diversity. When the two groups of mice were co-housed, their microbiota was similar, leading to increased mice survival that were formerly more probably to die (Fay et al., 2019). Similar sepsis experiments have demonstrated that a decrease in microbiota diversity can have an impact on animal mortality (Chen et al., 2018b). These findings show that damage to the intestinal microbiota is strongly associated with and subsequent progression of sepsis, and further emphasize the importance of the intestinal microbiota in sepsis. Based on current research, it is unclear how intestinal microbiota disruption prior to sepsis onset affects sepsis outcomes, but at least it can be determined that intestinal microbiota is one of the factors regulating systemic sepsis response; more research is needed to explore this mechanism in the future.

### 2.2 Sepsis worsens intestinal microbiota disruption

Some studies suggest the intestinal microbiota composition and function in sepsis patients is markedly disrupted compared to healthy individuals by employing multi-omics analysis (Liu et al., 2019; Yang X. et al., 2021). The disturbed intestinal microbiota lost the function of protecting the host, which may further lead to a serious response to sepsis and affect the results of sepsis treatment (Niu and Chen., 2021). When sepsis occurs, specific obligate anaerobes (e.g., *Bacteroidetes, Firmicutes*), which are usually predominant in healthy individuals, are frequently lost, and taxa with usually low levels of *Proteobacteria* (e.g., *Escherichia coli*, *Klebsiella pneumoniae*) “flowering” (Miller et al., 2021). Compared with healthy volunteers, the total number of anaerobic bacteria (including *Bifidobacterium* and *Lactobacillus*) has markedly decreased in systemic inflammatory response syndrome (SIRS) patients (Shimizu et al., 2011). One study found a significant decrease in intestinal microbiota diversity in sepsis patients, including a significant increase in abundance of inflammatory related microbes and pathogenic species (e.g. *Enterococcus spp.*). It also found a positive correlation between the abundance of *Enterococcus* species (OTU46) and sepsis mortality (Agudelo-Ochoa et al., 2020). In a study that included 12 intensive care unit (ICU) patients, the results were assessed using the ratio of *Bacteroides*/*Firmicutes* (B/F ratio), which found extreme changes in the B/F ratio in five of the six patients who died, suggesting a significant imbalance in the intestinal microbiota was strongly associated with patient prognosis (Ojima et al., 2016). It is evident that sepsis will aggravate the disruption of the intestinal microbiota, which can negatively affect sepsis outcomes.

In addition, the intestinal micro-ecology contains the normal flora of the gut and its environment, but also the metabolites produced by the intestinal microbiota (Chen et al., 2019). The metabolic products of the intestinal microbiota act in multiple ways to influence human health and disease (Connors et al., 2019). These metabolic products, such as short-chain fatty acids (SCFAs), are thought to be beneficial to their hosts (Anand et al., 2016). Compared to healthy individuals, sepsis patients showed a significant decrease in fecal SCFAS, with the change lasting 6 weeks (Yamada et al., 2015). Maintaining SCFAs concentrations also has a non-negligible effect on disease recovery due to its ability to protect intestinal barrier integrity and anti-inflammatory properties.

### 2.3 Intestinal microbiota modulates sepsis progression

#### 2.3.1 Relationship between intestinal microbiota and intestinal barrier

**FIGURE 1:**
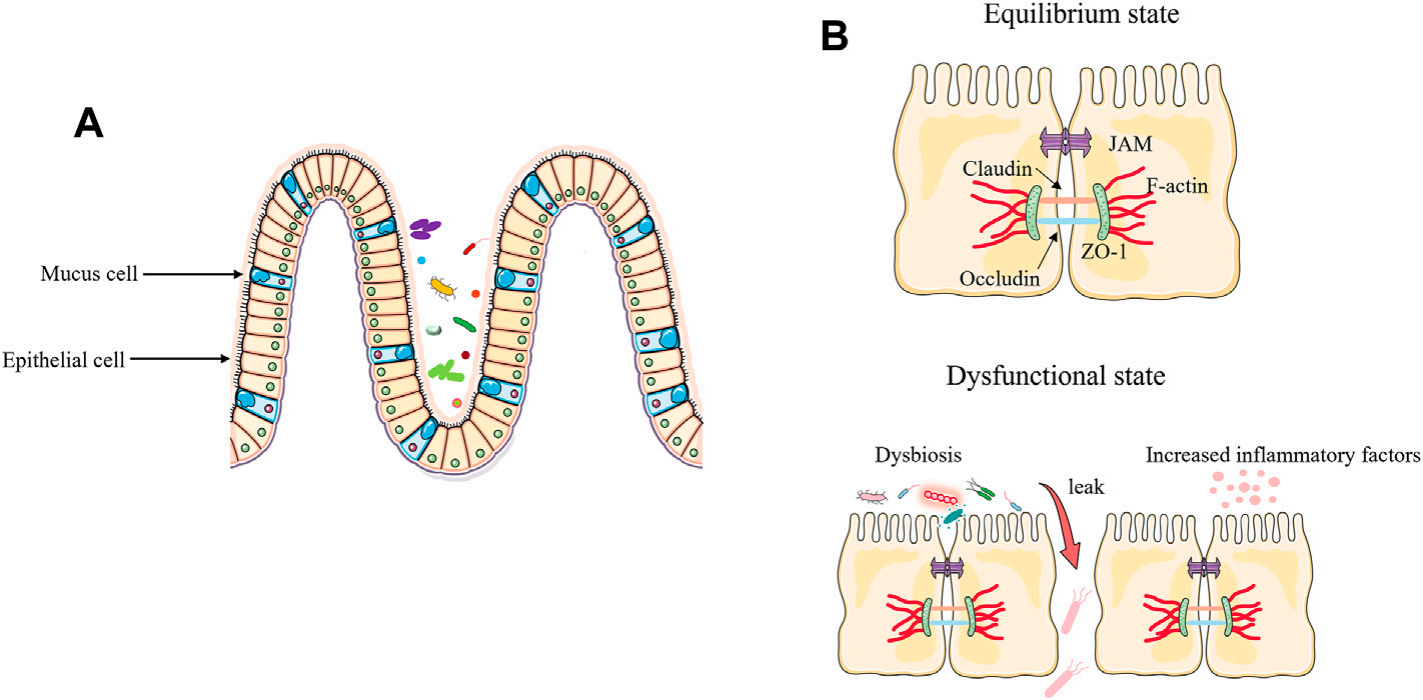
Disruption of the intestinal barrier and the function of the microbiota in sepsis. **(A)** Under healthy conditions, the intestinal epithelium consists of a single layer of intestinal epithelial cells (IECs), which is an important protective physical barrier that maintains intestinal homeostasis. **(B)** During sepsis, excessive release of inflammatory factors and loss of tight junction of epithelial cells leads to bacterial translocation and exacerbates the progression of sepsis.

With trillions of bacteria living in the intestinal tract, the integrity of the intestinal barrier is critical to a healthy status. The intestinal epithelium represents a key protective barrier, keeping pathogens at bay while ensuring the entry of beneficial substances (e.g., nutrients, products of symbiotic bacteria) (Krawczyk et al., 2018; Singh et al., 2019). The composition of tight junction includes occludin, claudins, zonula occludens-1 (ZO-1), junctional adhesion molecules, and many other types of proteins that provide a crucial primary barrier for intestinal space (Yoseph et al., 2016). The onset of sepsis destroys the intestinal barrier, causing an imbalance in the intestinal microbiota and resulting in bacteria migration from the intestinals (Sun et al., 2020). During sepsis, increased inflammatory cytokines can cause impairment of the intestinal barrier. In the clinically relevant models of sepsis, increased levels of cytokines can regulate the protein expression of claudin 2, claudin 5, junctional adhesion molecule-A (JAM-A), occludin, and ZO-1, resulting in increased gut permeability (Yoseph et al., 2016). In addition, myosin light chain kinase (MLCK), one of the signaling proteins involved in regulating intestinal barrier integrity, is positively associated with tumor necrosis factor alpha (TNF-α), and TNF-α promotes phosphorylation of MLCK, leading to disruption of tight junction and inducing loss of the epithelial barrier (Andrews et al., 2018; Zhang et al., 2021). Secondly, changes in the intestinal microbiota can also act on the intestinal barrier, mainly by down regulating the tight junctions between intestinal cells, leading to enhanced gut permeability (Hu et al., 2017; Wei et al., 2020). During sepsis pathogenesis, “beneficial” anaerobic bacteria families, like *Lachnospiraceae* and *Ruminococcaceae*, are lost and their absence can further impair the function of intestinal epithelial cells (Haak et al., 2018) (Figure 1).

#### 2.3.2 Intestinal microbiota influences the immune response during sepsis

From an immunological point of view, sepsis is closely associated with immune response disorder, with key factors including a systemic inflammatory response and disordered immune regulation (Chen and Wei, 2021). Intestinal microbiota are involved in the metabolism of substances and regulate the development and homeostasis of the immune system (Yin et al., 2017), while the immune system can recognize commensal bacteria and respond appropriately to potentially harmful pathogens (Leal-Lopes et al., 2015). Intestinal microbiota plays a significant role in fighting systemic infections by inducing the production of protective immunoglobulin G (IgG) (Zeng et al., 2016), IgA (Macpherson et al., 2015), and antimicrobial protein (Gallo and Hooper, 2012). The intestinal microbiota serves as an important regulator for the host immune system (Shi et al., 2020). The findings of Fay et al. suggested that the microbiome plays an important role in sepsis survival and host immune response (Fay et al., 2019). Microbiota, as a source of peptidoglycan, systematically stimulates the innate immune system and enhances the ability of bone marrow-derived neutrophils to kill pathogens (Clarke et al., 2010). Neutrophils are a critical component of innate immunity, Zhang et al. found that the microbiota gradually leads them to become more functionally active (Zhang et al., 2015). The intestinal microbiota induces local immune responses, and intestinal symbiotic gram-negative bacteria induces IgG responses and confer protection against systemic (Zeng et al., 2016).

The metabolic by-products of intestinal microbiota are not only a vehicle for communication through which the intestinal microbiota communicates with the immune system but are also one of the factors in influencing the immune system (Arpaia et al., 2013). Intestinal microbiota-derived SCFAs, primary vehicles for host-microbiota interactions in the gut, induce metabolic and transcriptional changes in macrophages, thereby enhancing their bactericidal functions (Schulthess et al., 2019). In addition, SCFAs can increase macrophage bacterial clearance by upregulating LAMTOR2 (Wu et al., 2020).

#### 2.3.3 Intestinal microbiota influence organ dysfunction during sepsis

Among sepsis-induced organ dysfunctions, including lung, liver, kidney, and brain injuries, are associated with microbiota disruption. Here, we use acute lung injury (ALI) as an example. The lung is one of the most vulnerable target organs in the pathogenesis of sepsis (Chen and Billiar, 2020). Acute respiratory distress syndrome (ARDS) is the most common organ dysfunction in COVID-19 (Karakike et al., 2021). Also, epidemiological reports indicate an upward trend in the incidence of ALI/ARDS due to sepsis is on the rise, with mortality rates of up to 70% (Zhao et al., 2016b; Chen H. et al., 2019). In a study, a mouse model of ALI caused by lipopolysaccharide (LPS) was established, and it was concluded that the pathological transformation of the ALI microbiota was caused by a group of congenital opportunistic pathogens that thrived in the inflamed lung environment, independent of external infectious factors (Poroyko et al., 2015). New research shows that the lung microbiota is rich in intestinal microbiota, both in animal models of septic ARDS and in patients with septic ARDS (Dickson et al., 2016). The above studies suggest that the intestinal microbiota is closely related to lung injury. Current studies have shown that typical intestinal microbiota (e.g., *Bacteroidetes* and *Enterobacteriaceae*) has been detected in the lungs of severe patients, suggesting that bacterial migration and subsequent exacerbation of the inflammatory response may be one of the potential pathogenic mechanisms of ARDS (Siwicka-Gieroba and Czarko-Wicha, 2020). Moreover, the intestinal microbiota can activate pulmonary oxidative stress and mediates lung damage by modulating the TLR4/NF-κB signal pathway (Tang et al., 2021).

**FIGURE 2:**
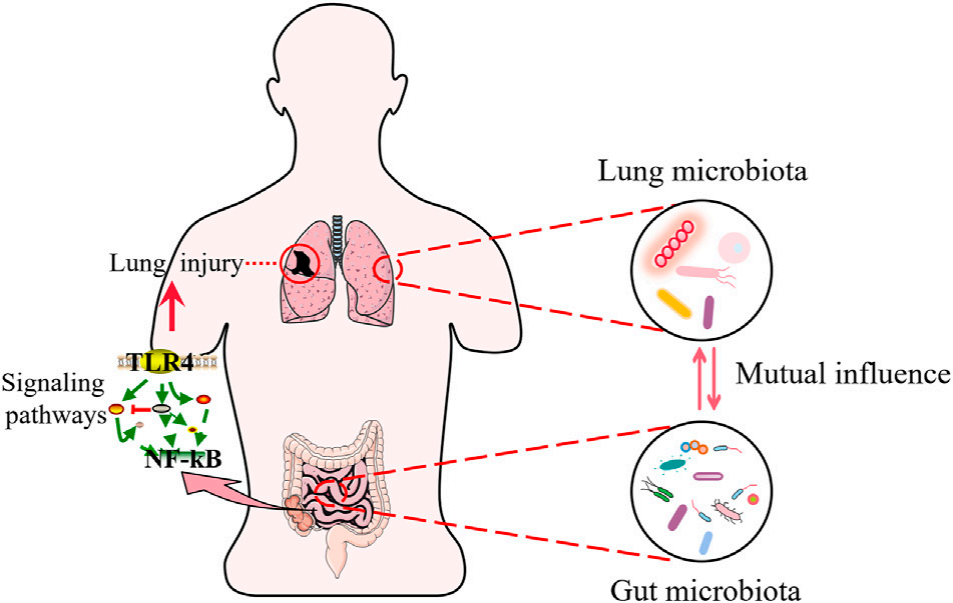
Sepsis and lung injury. Intestinal microbiota interact with lung microbiota during sepsis. The gut microbiota regulates signaling pathways and participates in sepsis induced lung injury.

In addition, the intestinal microbiota can also have an impact on the lung microbiota. Although traditionally considered sterile, the lung has been shown to contain a complex microbial community known as the lung microbiota, according to recently published investigations (Seixas et al., 2021). Comparing the microbiota in bronchoalveolar lavage (BAL) of healthy volunteers and patients with lung disease, major differences were found in the structure of the bacterial (Dickson et al., 2014). A recent clinical study has suggested that ARDS caused by sepsis is linked to a significant dysbiosis in the patient’s lung microbiota (Schmitt et al., 2020). The intestinal associated *Bacteroides* OTU, one of the most abundant components of intestinal microbiota, was found in the lungs of ARDS patients, but not in healthy controls (Dickson et al., 2020). On the contrary, changes in the lung microbiota can also influence the intestinal microbiota (He et al., 2017). LPS-induced ALI causes lung bacteria to metastasize into the blood and disrupts the bacteria composition of the cecum, causing a dramatic increase in bacterial numbers (Sze et al., 2014) (Figure 2).

## 3 Traditional Chinese Medicine and sepsis

### 3.1 Role of TCM in the treatment of sepsis

The pathogenesis of sepsis is complex and includes multiple aspects such as over-activation of the inflammatory response, immune dysfunction, disorder of blood coagulation and tissue damage. From the perspective of Western medicine, sepsis is a systemic inflammatory response caused by infection (Singer et al., 2016), and antibiotics are one of the most commonly used drugs. In a survey on drug-induced liver injury (DILI) related adverse drug reaction (ADR) (L-ADR) in China, it was found that in all L-ADR reports, TCM accounted for 4.5%, conventional drugs (including chemical and biological drugs) accounted for 95.5%, and antibiotics (including antitubercular agents) accounted for the highest proportion (Wang J. et al., 2022). However, TCM has a unique advantage in this area. A systematic review summarized TCM with potential anti-DILI effects, such as glycyrrhetinic acid, baicalin, and ginsenoside Rg1 (Sun et al., 2022), which also play a certain role in the treatment of sepsis. From the perspective of TCM, it is generally accepted that sepsis belongs to the TCM “febrile diseases” category, mainly based on the main theories such as “shanghanlun” and “wenrelun” (Lin S. J. et al., 2013). Several meta-analyses of clinical studies have shown that herbal medicine has a non-negligible role in the treatment of sepsis (Liang et al., 2015; Wen et al., 2021). Therefore, we summarized the TCM commonly used in sepsis treatment, mainly from the following five types of introduction: 1) Chinese patent medicine, such as Xuebijing injection, Shenfu injection, and Re-Du-Ning injection; 2) Traditional Chinese Medicine compound, such as Bai-Hu-Tang, Dachengqi decoction, and Huanglian Jiedu Decoction; 3) Couplet medicines, such as Coptidis Rhizoma - Euodiae Fructus drug pair; 4) Single herbal medicines, such as borneol, cordyceps sinensis, and *Rhizoma Coptidis*; 5) Single ingredients, such as Tanshinone IIA, emodin, crocin.

#### 3.1.1 Chinese patent medicine

Xuebijing injection (XBJ) was approved by the CFDA (China Food and Drug Administration) in 2004 for the treatment of sepsis and MODS (Chen et al., 2018a). In April 2020, XBJ was approved by the NMPA (National Medical Products Administration) for use as a treatment for COVID-19. XBJ plays an anti-sepsis protective role by reducing inflammatory factor release and modulating the differentiation of immune cells (Tregs and Th17) (Chen X. et al., 2018). XBJ inhibits the release of pro-inflammatory factors, promotes the production of anti-inflammatory factors, and modulates the GSK-3β pathway to alleviate CLP induced septic liver injury (Cao L. et al., 2021). Furthermore, antibiotic resistance is a pressing issue to be solved in the current treatment of sepsis, such as methicillin-resistant *Staphylococcus aureus* (MRSA), which is resistant to multiple antibiotics. TCM and its active ingredients have the potential to block antibiotic-resistant bacteria and provide a new treatment idea to reverse antibiotic resistance (Su et al., 2020). In a mouse model of MRSA-induced sepsis, XBJ plays a role in the treatment of drug-resistant bacterial infections by regulating the inflammatory response and inhibiting the Pam3CSK4 signal pathway (Li T. et al., 2020). This provides a preliminary basis for TCM to solve antibiotic resistance. At present, most studies have focused on clarifying the potential material basis and mechanism of XBJ in the treatment of sepsis. The study found that the main components of XBJ in the treatment of sepsis-induced cardiac dysfunction are paeoniflorin and hydroxysafflor yellow A, and its potential target may be related to NF-κB, TNF-α, and CXCL2 (Wang et al., 2021). Three components of Safflower have protective effects on LPS-induced ALI, and the potential mechanism may be related to inhibiting Raf/MEK/ERK pathway and the formation of neutrophil external traps (NET) (Wang Y. P. et al., 2020). In addition, the components of Ligusticum Chuanxiong hort may protect LPS-induced ALI by reducing NET formation (Zha et al., 2021). Similarly, Re-Du-Ning injection (RDN) plays the same role through this pathway and inhibits the MAPK pathway (Yang C. et al., 2021). RDN includes three herbal medicines, namely Artemisia annua, Honeysuckle and Gardenia gardenia (Wang Z. Y. et al., 2022). Shenfu injection may reduce LPS-induced myocardial apoptosis by inhibiting inflammatory response and MEK/ERK pathway activation (Chen et al., 2020b).

#### 3.1.2 Traditional Chinese Medicine compound

Traditional Chinese medicine compound plays an important role in TCM. It attaches importance to the compatibility of drugs and has been proved by most clinical practice in sepsis treatment. Bai-Hu-Tang, composed of Gypsum Fibrosum (Shigao), *Anemarrhena asphodeloides* Bunge (Zhimu), *Glycyrrhiza glabra* L. (Gancao), and Nonglutinous rice (Gengmi, polished seed of *Oryza sativa* L. in Gramineae), plays a certain role in the treatment of sepsis, improves the survival rate and reduces the level of inflammatory factors (Lin C. J. et al., 2013); at the same time, it also has a protective effect on the immune inflammatory damage caused by LPS (Zhang et al., 2013). Dachengqi decoction (DCQD) can relieve sepsis related ALI, regulate capillary permeability, reduce pulmonary edema, and inhibit inflammatory response, which is strongly associated with the TLR4/NF-κB signaling pathway (Hu et al., 2019). DCQD, which is composed of *Rheum palmatum* L. (Dahuang), *Citrus aurantium* L. (Zhishi), *Magnolia officinalis* Rehder and E.H.Wilson (Houpo), and Natrii sulfas (Mangxiao), have been confirmed to regulate gastrointestinal motility in MODS rats, possibly by repairing and protecting the enteric nervous system (Xie et al., 2015). Huanglian Jiedu Decoction (HLJDD) has a protective effect on septic rats. On the one hand, it can relieve excessive inflammatory response, and on the other hand, it can regulate immune response (Wei et al., 2013). Furthermore, HLJDD has a protective effect on sepsis related acute kidney injury (AKI), reduces oxidative stress and improves energy metabolism disorders in septic mice, which may be related to the activation of Akt/HO-1 signaling pathway (Li et al., 2017). Qingwen Baidu Decoction (QBD) has a remarkable anti-inflammatory effect and can be used to treat sepsis (Yu et al., 2014). In addition, QBD can reduce the degree of pulmonary edema in rats and prevent ALI caused by sepsis (Zhang et al., 2017). Dahuang Fuzi Decoction, which is composed of *Rheum palmatum* L. (Dahuang), *Aconitum carmichaeli* Debeaux (Fuzi), and *Asarum heterotropoides* F.Schmidt (Xixin), can improve the survival rate of sepsis rats and reduce the inflammatory response and intestinal barrier injury (Liu F. et al., 2020).

#### 3.1.3 Couplet medicines

Couplet medicines are the most basic and simplest form of compatibility of TCM, which is the bridge between a single herb and traditional Chinese medicine compound (Wang et al., 2012). As the smallest unit of TCM compatibility, the couplet medicine is composed of two relatively fixed herbs. Coptidis Rhizoma (Huanglian, CR) and Euodiae Fructus (Wuzhuyu, EF) is a well-known couplet medicines. Current research shows that, when the compatibility ratio of CR and EF is 6:1, it can inhibit the inflammatory response and reverse the lung injury caused by sepsis, possibly through the JAK1-STAT3 signaling pathway, and 7 components can be detected in rat serum, respectively: berberine, palmatine, jatrorrhizine, coptisine, evodin, chlorogenic acid, evodiamine (Yin et al., 2021).

#### 3.1.4 Single herbal medicines

Cordyceps sinensis has a protective effect on LPS induced intestinal injury, which can promote cell proliferation and restore intestinal barrier function (Gu et al., 2015). *Rhizoma Coptidis* protects against sepsis related AKI by acting on HO-1, NOS2 and PPAR α and other 17 target proteins (Zheng et al., 2021). *Lonicera japonica* Thunb. can improve immune dysfunction caused by sepsis through inhibiting immune cell apoptosis (Kim et al., 2015). Borneol is protective against sepsis-related brain injury, reducing neuroinflammation in the brain and protecting neurons and microglia, with potential mechanisms closely related to the NF-κB and MAPK pathways (Wang et al., 2019). *Panax notoginseng* reduces the inflammatory response of CLP-induced sepsis by inhibiting the NF-κB signaling pathway (Shou et al., 2022). North American ginseng improves LPS-induced cardiac dysfunction in mice with endotoxemia by inhibiting the NOX2-ERK1/2-TNF-α signaling pathway, suggesting its potential for preventing sepsis (Wu et al., 2016). Most of these herbal medicines have anti-inflammatory and immunomodulatory properties, so they are used to study the treatment of sepsis.

#### 3.1.5 Single ingredients

Pristimerin can ameliorate neuronal injury in septic brain injury mice, mainly by regulating PI3K/Akt signaling pathway (Xue et al., 2021). Tanshinone IIA has a neuroprotective effect on sepsis mice, and in addition to its anti-inflammatory effects, its mechanism may be related to inhibiting the activation of astrocytes and microglia (Xiong et al., 2019). Cinnamyl alcohol protected septic mice by inhibiting the NLRP3 inflammatory pathway (Zou et al., 2022). Similarly, emodin attenuates LPS-induced myocardial injury through this pathway (Dai et al., 2021). Glycyrrhizic acid can prevent septic AKI and septic ALI, mainly by inhibiting inflammatory response, reducing oxidative stress damage and reducing cell apoptosis (Zhao et al., 2016a; Zhao et al., 2016b). In addition, glycyrrhizin alleviates ARDS caused by sepsis by inhibiting the HMGB1/TLR9 pathway (Gu et al., 2022; Zhao et al., 2017). Crocin is considered as a potential drug for the treatment of sepsis, because it shows significant anti-inflammatory and anti-apoptotic effects, primarily associated with MAPK/NF-κB and Bax/Bcl-2 signaling pathways (Gao et al., 2022). Studies have shown that ginsenoside Rg3 can alleviate cell and organ damage caused by sepsis, mainly acting on the AMPK signal pathway and alleviating mitochondrial dysfunction caused by sepsis (Xing et al., 2017).

**FIGURE 3:**
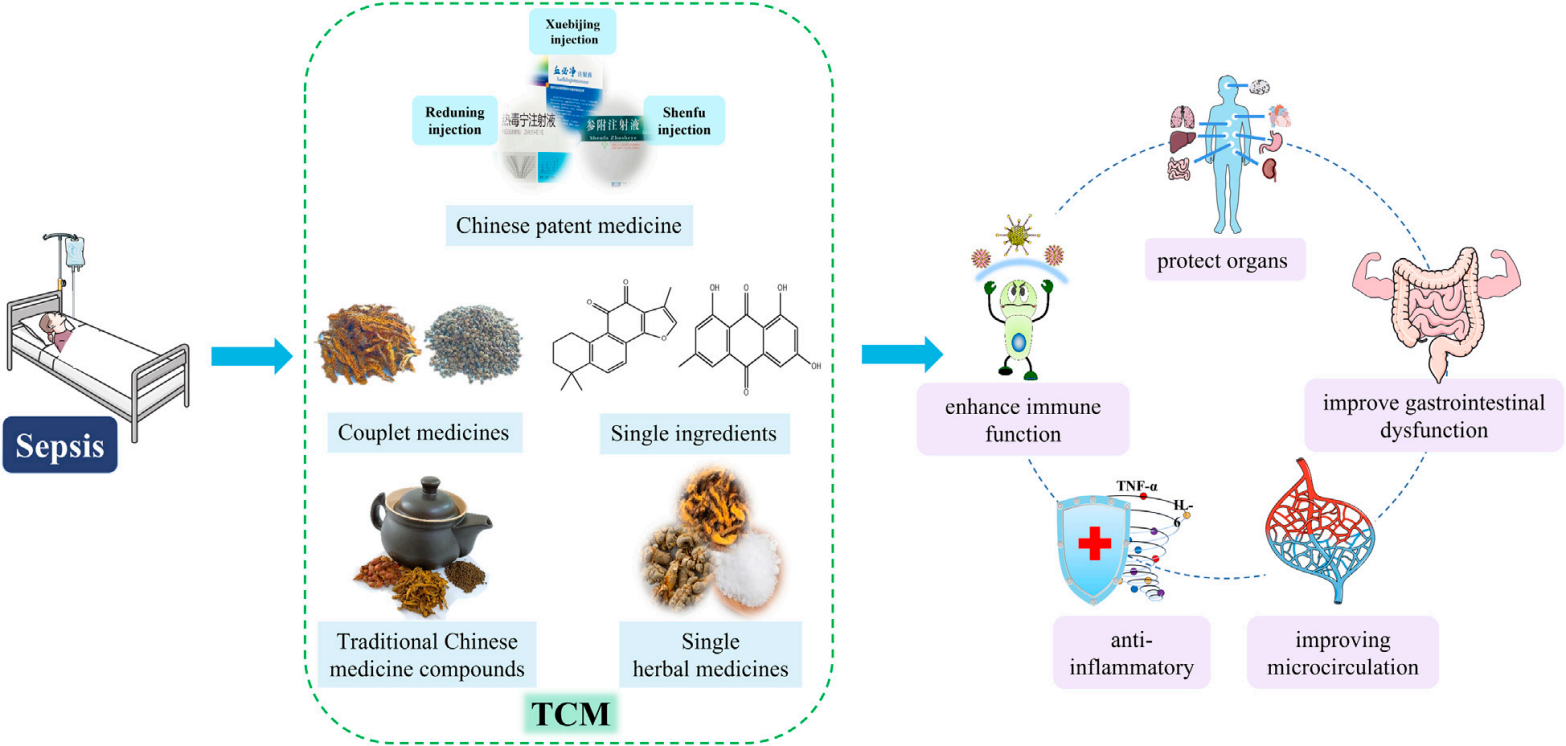
Therapeutic effects of TCM on sepsis. TCM, includes Chinese patent medicine, traditional Chinese Medicine compound, couplet medicines, single herbal medicines and single ingredients, treats sepsis in a variety of ways, such as anti-inflammatory, improving gastrointestinal disorders, enhancing immune function, improving microcirculation, and protecting organs.

These studies show that TCM has a certain role in the treatment of sepsis. These drugs have many effects in the treatment of sepsis, such as anti-inflammatory, improve gastrointestinal dysfunction, enhance immune function, protect organs and so on (Figure 3).

### 3.2 Mechanisms of TCM alleviating sepsis *via* the intestinal microbiota

**FIGURE 4:**
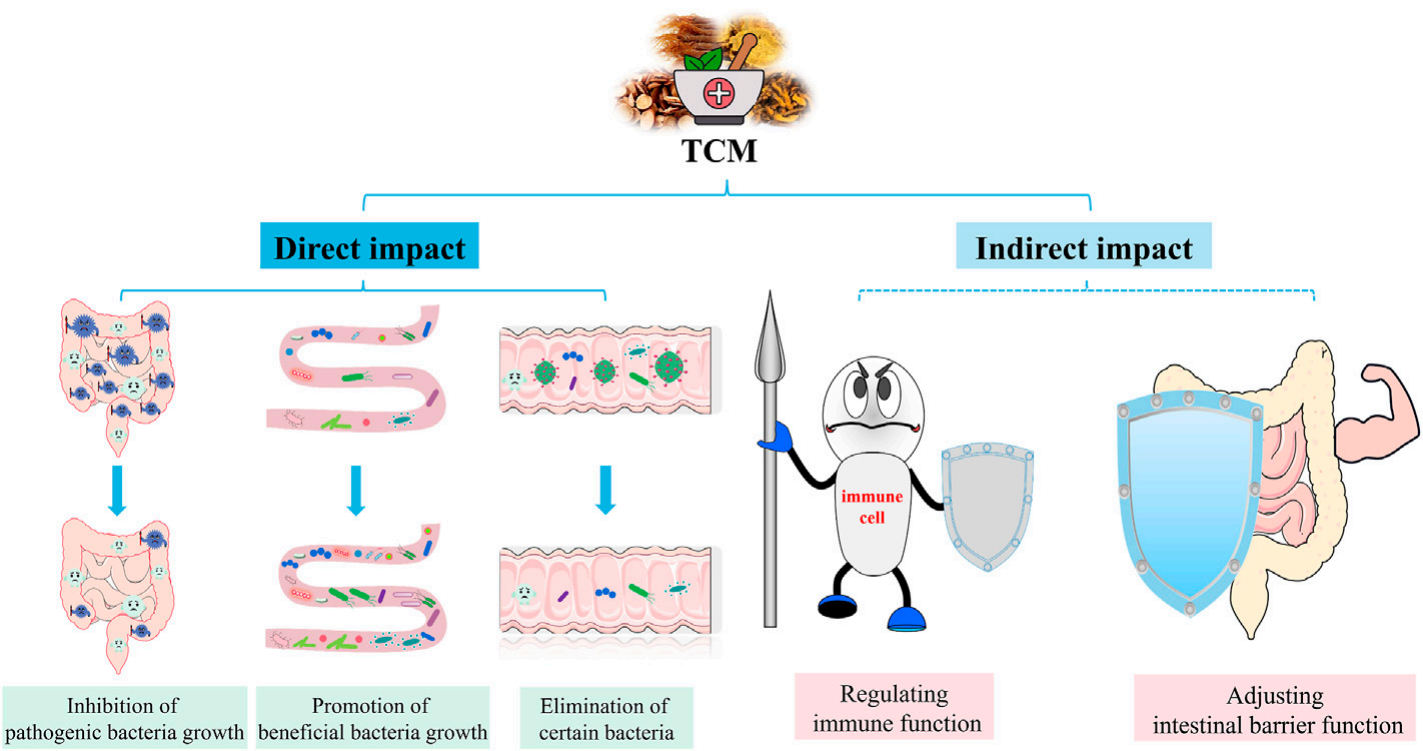
Effects of TCM on intestinal microbiota. TCM can directly and indirectly regulate the intestinal microbiota to relieve sepsis. Direct effects include the promotion, suppression and elimination of specific groups of bacteria. Indirect effects include regulation of intestinal barrier function and immune homeostasis.

Antibiotics are essential for the treatment of sepsis (Cao et al., 2019). It is known to fight pathogens, but can also inhibit symbiotic bacteria, disrupt intestinal microbiota and affect host health (Maier et al., 2021). A randomised controlled study showed that antibiotic treatment significantly reduced intestinal microbiota diversity in all subjects (Lankelma et al., 2017). Similar findings were also found in sepsis animal models. The use of antibiotics disrupts the intestinal microbiota, leading to an increase in pathogenic bacteria (Han et al., 2021). In addition, an observational study showed that after antibiotic use in patients with sepsis, the intestinal microbiota was dominated by *Klebsiella* or *Enterococcus*, which may further lead to secondary infections (Mu et al., 2022). Taken together, antibiotics affect the intestinal microbiota and may have implications for subsequent treatment, and TCM has unique advantages in this regard. In recent years, researchers have combined intestinal microbiota with TCM, with a view to investigating the mechanisms of TCM in the preventing of sepsis. The interaction between TCM and intestinal microbiota, both direct and indirect, can cause structural changes in the intestinal microbiota, as well as changes in metabolites of the intestinal microbiota, which have an impact on the development and prognosis of sepsis. TCM is generally administered orally, and they can be in direct contact with the intestinal microbiota and therefore affecting the intestinal microbiota composition. These direct effects include promotion, inhibition, and elimination. Flavonoids, polysaccharides and saponins in TCM can act as prebiotics and facilitate growth of certain intestinal microbiota (Feng et al., 2019). In addition, some TCM ingredients could inhibit or kill microorganisms. The indirect impact of TCM on the intestinal microbiota involves adjusting intestinal barrier function and regulation of immune homeostasis (Figure 4). Therefore, we summarize the treatment of sepsis with TCM from the following three aspects.

#### 3.2.1 Regulation of intestinal microbiota structure

TCM can play a role in disease prevention by regulating the dysfunctional intestinal microbiota structure under disease conditions. Sini decoction (SND), consisting of *Aconitum carmichaeli* Debeaux, *Zingiber officinale* Roscoe, and *Glycyrrhiza glabra* L., has been ameliorate sepsis symptoms *via* modulating the microbiota in the intestinal tract. At the genus level, SND can also promote the consumption of harmful bacteria (*Ruminococcaceae*, *Lachnoclostridium*, *Faecalibaculum* and *Oscillibacter*) in the faeces of pathological mice induced by CLP (Wang W. et al., 2020). The number of *Bacteroidaceae*, *ClostridiumXI* and *Parabacteroides* in the sepsis group was restored to normal after treatment with Xuanbai Chengqi Decoction (XBCQ) (Mu et al., 2021). A study showed that the intestinal microbiota of septic rats treated with probiotics and QingRe JieDu Decoction (QRD) had the same diversity and composition, suggesting that QRD reduced mortality in septic animals without damaging the characteristics of the intestinal microbiota (Cao H. et al., 2021). Sinomenine, an isoquinoline alkaloid, derived from *Sinomenium acutum* (Thunb.) Rehder and E.H.Wilson. One study found that sinomenine augmented the number of beneficial bacteria (such as *Prevotellaceae UCG-001*) and lessened the number of noxious bacteria (such as *Escherichia-Shigella*) to regulate gut homeostasis (Song et al., 2021). Treatment with emodin can restore the reduced diversity of intestinal microbiota caused by sepsis, as well as inhibit the growth of harmful bacteria that contribute to the development of sepsis, such as facultative anaerobes and *Proteobacteria*, and restore the number of beneficial bacteria, such as *Firmicutes* and *Bacteroidetes* (Zhang et al., 2022).

#### 3.2.2 Regulation of intestinal barrier

The intestinal barrier function plays an important role in maintaining the homeostasis of the body. The intestinal barrier function can effectively prevent the intestinal bacteria and their toxins from migrating to the tissue and organs and protect the body from endogenous microorganisms and their toxins. In addition to regulating the structure of the intestinal microbiota, TCM can also indirectly influence bacterial migration through the protection of the intestinal barrier. Shenfu injection (SFI) had a protective effect on the intestinal mucosa in rats with sepsis (Jin et al., 2019), significantly improving the disruption of tight junctions between intestinal epithelial cells (Xing et al., 2015). Before sepsis, pre-treatment with *Rheum palmatum* L. can promote the intestinal mucosal capillaries dilatation, protect capillary endothelial cells, and improve intestinal microcirculation in sepsis (Cui et al., 2014), and ameliorates intestinal permeability with septic patients (Fang et al., 2007). Moreover, emodin enhances the expression of ZO-1 and occludin, improves intestinal barrier function, and prevents bacterial translocation again (Zhang et al., 2022). Intervention with Jinzhi reduced the inflammatory factors (i.e., TNF-α, IL-1β, and IL-6) released by LPS-induced intestinal mucosal barrier damage and increased the expression of occludin and claudin-1 (Xu et al., 2020). Berberine can reduce intestinal damage caused by LPS, through increasing the activating of SOD and GSH-Px and inhibiting TLR4 and NF-κB in the ileum (Zhang et al., 2011). Furthermore, berberine can also induce Zrt-Irt-like protein 14 expression to protect the intestinal barrier in sepsis (He et al., 2019), and ameliorates sepsis-induced reduction in intestinal vascular barrier permeability caused by sepsis by modulating the ApoM/S1P pathway (Li Y. et al., 2020). Berberine has also been shown to protect the intestinal mucosal barrier by mediating Toll-like receptors (Li et al., 2015). Sinomenine therapy effectually reduces CLP-induced colonic damage and depletion of tight junction proteins (Song et al., 2021). In conclusion, TCM maintains the intestinal barrier effect to resist pathogenic microbial attack and inhibits the migration of bacteria in sepsis.

#### 3.2.3 Regulating immunity

Sepsis is closely related to disordered immune response. Therefore, during the treatment of sepsis, it is very significant to strengthen the body’s autoimmunity and adjust the immune balance. The immunomodulatory effect of TCM can’t be ignored, which is not only reflected in single herbal medicines or single ingredients, but also in traditional Chinese medicine compound. Numbers of white blood cells, neutrophils, and lymphocytes were decreased significantly in sepsis, which can be improved by HLJDD, indicating that HLJDD can improve bacteria-killing function and enhance immunity (Lv et al., 2017). Sepsis leads to a reduction in T helper (Th) cells, which treatment with *Astragalus* polysaccharides can improve and further stimulate a balanced Th1/Th2 response to alleviate immune suppression of sepsis (Hou et al., 2015). In LPS-induced macrophages, shionone (SHI) had an opposite effect on the expression of M1 and M2 polarization biomarkers, mainly by inhibiting M1 and promoting M2, possibly *via* the ECM1/STAT5/NF-κB pathway. Moreover, SHI could promote macrophage function through increasing the levels of GM-CSF (Song et al., 2022).

## 4 Conclusion and prospect

Sepsis is mainly treated with antibiotics and the following problems may exist in the use. First, antibiotic use may cause DILI, which further exacerbates sepsis-induced organ dysfunction. Second, the use of antibiotics can cause intestinal microbiota disturbance. The intestinal microbiota dysbiosis is a significant hidden risk factor of sepsis. Additionally, the intestinal microbiota is involved in the progress of sepsis and may also lead to organ failure. Therefore, enhancing the gut barrier function and improving the immune system by regulating the intestinal microbiota is expected to be one of the treatment options for sepsis. TCM has been proved to recover microbiota homeostasis, ameliorate intestinal barrier integrity, and modulate immune reactions. At present, from the point of view of intestinal microbiota, some progress has been made in the comprehension and application of TCM. Later, research should keep a watchful eye on the causal relationship among TCM, intestinal microbiota, metabolites and treatment results, and then better apply it to clinical practice. In addition, with the development of high-throughput-omics technologies, metabolomics has been an ideal tool for study of TCM-intestinal microbiota interactions. The integrated analysis of intestinal microbiota and metabolomics allows the identification, quantification and characterization of certain compounds produced by gut microbes, promising further understanding of the possible mechanisms of TCM for the therapy of disease. Finally, antibiotic resistance is an urgent problem to be solved. TCM has a potential role in this aspect and is expected to reverse antibiotic resistance, which also requires more in-depth research. At the same time, TCM also has certain limitations in terms of treatment, such as the lack of basic material research. Thus, a joint multidisciplinary effort will be required to elucidate the material basis of herbal medicine for sepsis. In addition, TCM has certain advantages in the treatment of sepsis and can regulate intestinal microbiota. Therefore, subsequent studies should combine intestinal microbiota with TCM in order to reveal the possible mechanism of TCM in treating diseases.

## Abbreviations

ADR, adverse drug reaction; ALI, acute lung injury; ARDS, acute respiratory distress syndrome; AKI, acute kidney injury; BAL, bronchoalveolar lavage; CDI, *Clostridium difficile* infection; CLP, cecal ligation and puncture; CFDA, China food and drug administration; DILI, drug-induced liver injury; GSH-Px, glutathione peroxidase; IgA, immunoglobulin A; IgG, immunoglobulin G; IL-10, interleukin-10; IL-1β, interleukin-1β; IL-6, interleukin-6; JAM-A, junctional adhesion molecule-A; LPS, lipopolysaccharide; MLCK, myosin light chain kinase; MODS, multiple organ dysfunction syndrome; NF-κB, nuclear factor kappa-B; NMPA, national medical products administration; SCFAs, short-chain fatty acids; SIRS, systemic inflammatory response syndrome; SOD, superoxide dismutase; TCM, traditional Chinese Medicine; Th, T helper; TLR4, Toll-like receptors 4; TNF-α, tumor necrosis factor alpha; ZO-1, zonula occludens-1.
